# Feasibility of conducting HIV combination prevention interventions in fishing communities in Uganda: A pilot cluster randomised trial

**DOI:** 10.1371/journal.pone.0210719

**Published:** 2019-03-27

**Authors:** Monica O. Kuteesa, Helen A. Weiss, Andrew Abaasa, Stephen Nash, Rebecca N. Nsubuga, Rob Newton, Janet Seeley, Anatoli Kamali

**Affiliations:** 1 MRC/UVRI and LSHTM Uganda Research Unit, Entebbe Uganda; 2 London School of Hygiene and Tropical Medicine, London United Kingdom; 3 MRC Tropical Epidemiology Group, London School of Hygiene and Tropical Medicine, London United Kingdom; 4 International AIDS Vaccine Initiative, Nairobi, Kenya; The Chinese University of Hong Kong, HONG KONG

## Abstract

**Objective:**

We assessed feasibility of an HIV-combination-prevention trial among fishing communities in Uganda.

**Design:**

Cluster randomised trial in four fishing communities on Lake Victoria, Uganda. Two intervention communities received a combination-prevention-package (behaviour change communication, condom promotion, HIV testing, voluntary male medical circumcision and referral for anti-retroviral therapy if HIV-positive). All four communities received routine government HIV care services.

**Methods:**

Using household census data we randomly selected a cohort of consenting residents aged ≥18 years. A baseline sero-survey in July 2014 was followed by two repeat surveys in March and December 2015. We measured uptake of HIV prevention methods, loss-to-follow-up and HIV incidence, accounting for multistage survey design.

**Results:**

A total of 862 participants were enrolled and followed for 15 months. Participation was 62% and 74% in the control and intervention arms respectively; Overall loss to follow up (LTFU) was 21.6% and was similar by arm. Self-reported abstinence/faithfulness increased between baseline and endline in both arms from 53% to 73% in the control arm, and 55% to 67% in the intervention arm. Reported condom use throughout the study period was 36% in the intervention arm vs 28% in the control arm; number of male participants reporting circumsicion in both arms from 58% to 79% in the intervention arm, and 39% to 46% in the control arm. Independent baseline predictors of loss-to-follow-up were: being HIV positive, residence in the community for <1 year, younger age, living in an urban area, and being away from the area for >1 month/year

**Conclusions:**

Recruitment and retention of participants in longitudinal trials in highly mobile HIV fishing communities is challenging. Future research should investigate modes for locating and retaining participants, and delivery of HIV-combination prevention.

## Introduction

With major advances in HIV treatment and prevention, the number of new HIV infections among adults globally declined by an estimated 11%, from 3.4 million in 1996 to 1.8 million in 2017 [1]. However, HIV incidence remains high in many settings, including eastern and southern Africa. An estimated 43% of new HIV infections globally occur in this region [1]. In Uganda, the number of new HIV infections reduced by 63% from 140,000 in 2013 to 52,000 in 2016 [2]. Some studies have demonstrated the feasibility of achieving the UNAIDS 2020 target: 90% of all people living with HIV will know their HIV status, 90% of all people with diagnosed HIV infection will receive sustained antiretroviral therapy, 90% of all people receiving antiretroviral therapy will have viral suppression 90-90-90) [3,4].

Combination prevention strategies, particularly among key populations, are needed for a sustained impact on global HIV incidence, and to alleviate the economic burden in low- and middle-income countries [5]. Recent findings from hyper-endemic fishing communities show that over a five year period, scale-up of anti-retroviral therapy (ART) and voluntary medical male circumcision (VMMC) contributed to reduction of HIV incidence by approximately 54% (3.98/100 py to 1.61/100 py (adjIRR = 0.46; 95%CI: 0.27–0.80) [6]. Other ongoing combination prevention trials in eastern and southern Africa suggest that implementing effective HIV treatment and prevention interventions in high prevalence settings is feasible, and can be rapidly scaled to achieve the UNAIDS 90-90-90 target [7, 8].

Few studies have examined uptake of HIV combination prevention interventions in resource-poor, highly-mobile key population settings. In Uganda, this includes people living and working in fishing communities [9–13], where HIV prevalence and incidence is high (prevalence: 22%-37%; incidence: 3.3–6.7 cases/100 pyr [14–17]. In comparison, national HIV prevalence is 7.0% and incidence is <1/100 pyr [18, 19]. The high HIV incidence in the fishing communities is due to an interaction of multilevel factors including high levels of mobility, prolonged separation from spouses and families, inadequate social services [10, 19, 16, 20] peer norms that encourage hyper-masculinity and high-risk sexual behaviour [10, 15, 21]. Vulnerability to HIV is exacerbated by the ready availability of transactional sex, and access to daily disposable income in an overall context of poverty, and widespread alcohol and drug use [21]. Potential barriers to successful, sustained implementation of combination prevention in fishing communities include high mobility of fisher folk, widely-held myths and misconceptions about HIV infection and HIV prevention, and poor access to HIV prevention services [22–24]. Assessing the feasibility of conducting combination HIV prevention interventions in this population will guide design of prevention trials that are responsive to these barriers.

The aim of this paper is to present results of a pilot study assessing the feasibility of implementing HIV combination prevention interventions at community level in fishing communities in Uganda. We also examine recruitment and retention of study participants, and the feasibility of a larger trial.

## Materials and methods

### Study setting and design

**Fig 1:**
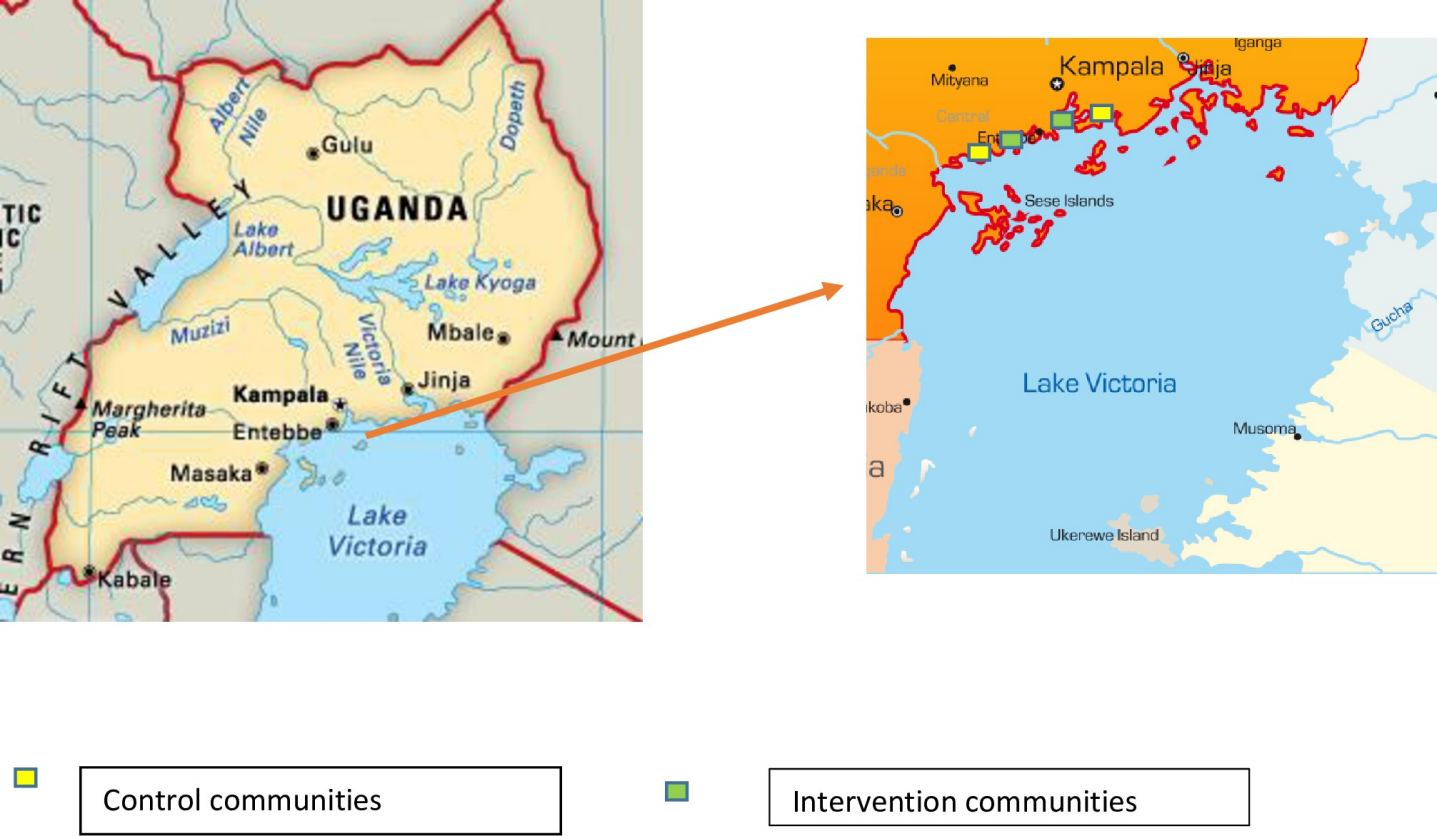
Map showing the intervention and control study region and communities.

The study was a pilot cluster-randomised trial (CRT), with four purposively selected clusters (study communities). Selection criteria were two rural, two urban geographically distant communities with a health facility where community members can access care. The clusters were fishing communities situated along Lake Victoria shores in Uganda, in three districts (Wakiso, Kampala, Mpigi). Within each urban or rural pair, one cluster was randomly assigned to receive the combination prevention package, while the other cluster served as a control. The two urban communities were located approximately 5Kms (for the intervention) and 18Kms (for the control) away from Kampala city center. The rural communities were located 62Kms (for the intervention) and 71Kms (for the control) away from Kampala city center (Fig 1).

A cohort of participants was followed within each community to assess uptake of the intervention strategies and loss-to-follow-up (LTFU). The eligibility criteria for clusters included a population size of ≥1000, access to a public health facility, absence of ongoing research and permission from local leaders to conduct research.

### Study procedures

From March to May 2014, we conducted a mapping exercise, and a household census of the four communities to form the individual-level sampling frame.

### Sample size

We aimed at sampling 350 participants in each community. This sample size would be sufficient to get reasonably precise estimates of the proportions who take up each component of the combination prevention package with a precision of approximately ± 5.5% if the level of uptake is about 50%. We recruited at least 100 HIV-uninfected males and 100 HIV-uninfected females in each community, allowing for LTFU and refusals. We conducted a census in March 2014. The census enumerated all adult residents and temporary residents including individuals who were away temporarily. e.g. for work or school, and regular visitors who stay at least one night such as fish traders. In each of the participating communities, the population was divided into six strata, stratified on gender and age (18–24 years, 24–34 years and ≥35 years). Sampling weights inversely proportional to the sampling probability of a participant were calculated for each strata, according to the target sampling probabilities. The sample weights were constant over the four study communities. The sampling fractions were based on the HIV prevalence found in the earlier study (16), and hence differed between age strata. The census showed that the average number of adults living in a household was five, so 70 households were randomly sampled per community to reach the required sample size.

### Community engagement

For all communities we involved the district and local leadership and community members in protocol development, implementation and dissemination of initial findings through district level meetings and two study specific community advisory boards.

To roll-out the intervention package, to facilitate uptake of interventions and to ensure compliance and retention for enrolled participants, 10 field workers were supported by four community members including village health team workers and local leaders in each study community. The field team spoke the local language and used education, entertainment and other information, education and communication materials to explain the intervention. The amount of work/effort required by the team to implement the intervention was not measured specifically. These would be important data to collect in any further testing of the intervention to inform the feasibility of roll-out of the package. To facilitate study implementation we used a strategic mix of facility and non-facility based strategies for both community engagement and delivery of interventions. This included setting up community hubs where all study procedures were conducted, use of entertainment, sports and social messages to deliver information, education and communication about HIV and HIV prevention, and training of local health workers and village health team members. This approach may be useful to achieving universal and potentially, equitable access to HIV prevention methods, in particular for communities that are geographically remote [25, 26], distant from heath facilities, and with highly mobile residents [27]. We provided STI treatments and treatments for other common illnesses to community members including children.

### Participant recruitment and follow-up

All participants in a selected household were invited to the community hub for screening for inclusion in the cohort. Inclusion criteria were age ≥18 years and resident in the community for over three months. To maximise the participation rate, three repeat visits, over a three month period were made to selected households with missing participants. Participants who met the eligibility criteria were given unique identification numbers and were provided with information about the study and invited to provide written informed consent. Ninety-six participants unable to read provided witnessed consent.

We conducted a baseline sero-survey among a population-based sample of adults aged ≥18 years, in July 2014. We conducted two repeat surveys with this same cohort at month 9 i.e. March 2015, and month 15 i.e. December 2015. To minimise contamination across study communities we selected geographically distant communities. Monitoring contamination was challenging because of lack of biometric evaluation.

### Public randomisation of study arms

In August 2014, a public randomisation ceremony was held in each pair of communities, attended by two representatives from each community. Community leaders tossed a coin to randomly determine which community within a pair would receive the intervention.

### Qualitative methods

We conducted formative qualitative studies to inform community engagement and study implementation, and to supplement feasibility data providing additional insight into how people in fishing communities experience uptake of HIV prevention methods, singly and in combination. Details are published elsewhere [28].

### Data collection procedures

Interviewer-administered data on socio-demographic factors, risk behaviour, and uptake of HIV prevention methods were collected using Android tablets. Following the interview and pre-test counseling, participants provided 5mls of venous blood for HIV rapid tests. Female participants provided urine samples for pregnancy tests. During follow-up, participants were scheduled to return to the community hub once every 6 months for repeat visits, which involved interviews on social demographics, risk behaviour, the uptake of selected HIV prevention methods, and HCT. Trial staff and village heath teams used previously-collected locator information to trace participants lost to follow up, making weekly calls and household visits over the four months of the survey.

### Laboratory methods

HIV rapid tests were conducted at the community hub using a pre-defined approved standard operating procedure and national testing algorithm [29]: Alere Determine HIV 1/2 whole blood assay (Alere medical, Chiba, Japan), STAT-PAK rapid test HIV 1/2 (Chembio diagnostic systems NewYork, U.S.A) and Unigold (Trinity Biotech, Wicklow, Ireland); HIV confirmatory tests were conducted in an accredited laboratory (Entebbe MRC/UVRI) using ELISA HIV Murex Diasori: Ref 9E25-02 UK and BIOKIT Bio Elisa HIV1/2 Ag/Ab, SPAI.

### Intervention implementation

We delivered community-wide interventions from October 2014 to February 2016.

#### Components of the intervention

Communities in the control arm received a standard HIV prevention package that was intermittently provided by the public health facilities including condoms, HIV testing and counselling, behaviour change communication, VMMC, and referral for ART. Communities in the intervention arm received both the standard of care package provided by existing public health facilities and the additional components provided by the study including:

- Community hub-based voluntary HIV testing and counselling (HCT) offered to all residents throughout the study period.
- Linkage to care: referring all participants diagnosed HIV-positive to the local primary health facility for HIV treatment and care. Pregnant women who were diagnosed HIV-positive were encouraged to access prevention of mother to child transmission (PMTCT) services.
- Voluntary medical male circumcision: VMMC was available to uncircumcised male residents through VMMC camps, and we encouraged men to present to locally available services for VMMC.
- Condom promotion: During community meetings, the household visits, the study team provided behavioural risk reduction counselling and offered a supply of free condoms.
- Screening for symptoms suggestive of sexually transmitted infections, with syndromic management and referral to the local health care facility for appropriate further management as necessary.

During the intervention implementation, we used a combination of innovative methods, at the facility and in the community, to maximise continuous community engagement. These activities included: involving community members in public randomisation, community mobilisation, and direct participation of community members in behaviour change communication through competitive sports, entertainment and health education and community advisory board meetings, outreaches to groups such as female sex workers, faith based organisations, primary and secondary schools. As part of the intervention, both participants and researchers explored and directly addressed HIV infection and HIV prevention related myths and misconceptions [28].

The main differences in activities between control and the intervention group activities is that public facilities did not offer VMMC and condom distribution or promotion routinely. During the study duration, to our knowledge, no surgical camps were conducted by any of the public health facilities. In addition, behaviour change communication activities delivered through public health facilities to the control arm were ad hoc, and often delivered at individual level at the health facility.

#### Timing and delivery of intervention

Between August 2014 and December 2015, the study team provided all the study components in the intervention arm except for VMMC, which was provided by a local not-for-profit VMMC-provision organisation.

Upon completion of follow-up in December 2015 to February 2016, the HIV combination prevention package was rolled-out to the control arms. In January 2016, following the new WHO guidelines, two Tetanus Toxoid Containing vaccines (TTCV) doses were provided four weeks apart, with the second dose provided between seven to 14 days, prior to VMMC [30, 31].

The staff in this study were of medical and social science backgrounds. The study team had extensive experience in conducting field work for surveys and large intervention studies in fishing communities and elsewhere. Additionally, prior to the start of the study, the team was trained in all study procedures and the study protocol using mock and pilot questionnaires and activities. They also underwent Good Clinical Practice training prior to the start of the study.

### Data management and analysis

#### Data management

The study database was developed using Microsoft Access. Data quality and integrity were ensured through the implementation of quality control procedures during the database programming. Queries generated by the data management team were directed to the clinical study staff.

#### Outcomes

Outcomes were the proportion of cohort participants who were lost-to-follow-up and self-reported use of HIV prevention methods. A participant was deemed lost-to-follow-up if he/she participated in the baseline survey but did not participate in either of the following two surveys.

#### Process measures

The following process measures were recorded in both study arms to evaluate the implementation and delivery of the interventions: HIV testing, respondents referred for HIV care, number of men reached with VMMC services, number of condoms distributed. We did not fully evaluate self-reported use of ART because we did not provide ART in the study, we referred participants for ART to other HIV care centres.

#### Statistical analysis

All analyses were carried out using Stata version 15. As this was a feasibility study, no formal hypothesis tests were performed for differences between intervention and control communities. Baseline characteristics, including HIV prevalence and socio-demographic characteristics of study participants were summarised by study arm. We investigated risk factors for loss-to-follow-up through a multivariable logistic regression model of baseline characteristics, retaining in the model any factors, which caused an increase in odds of greater than 25% (equivalent to a decrease of 20%).

We built a model to adjust for baseline values of HIV status, sex, residence (urban or rural), age group (18–24, 25–34, 35+), time living in the community (less than one year, more than one year), time away from the community per year (less than one month, more than one month) and working in a hotel or bar.

#### Ethics statement

The study was approved by the Uganda Virus Research Institute, the Uganda National Council of Science and Technology, and the London School of Hygiene and Tropical Medicine ethics committees. At the three scheduled visits to the community hub, we compensated participants for their time and transport costs (10,000 Uganda shillings ~3 USD per head and a snack for each sero-survey). Referrals to public health facilities were made for family planning, ART and other medical services that were not offered in the study.

## Results

**Fig 2:**
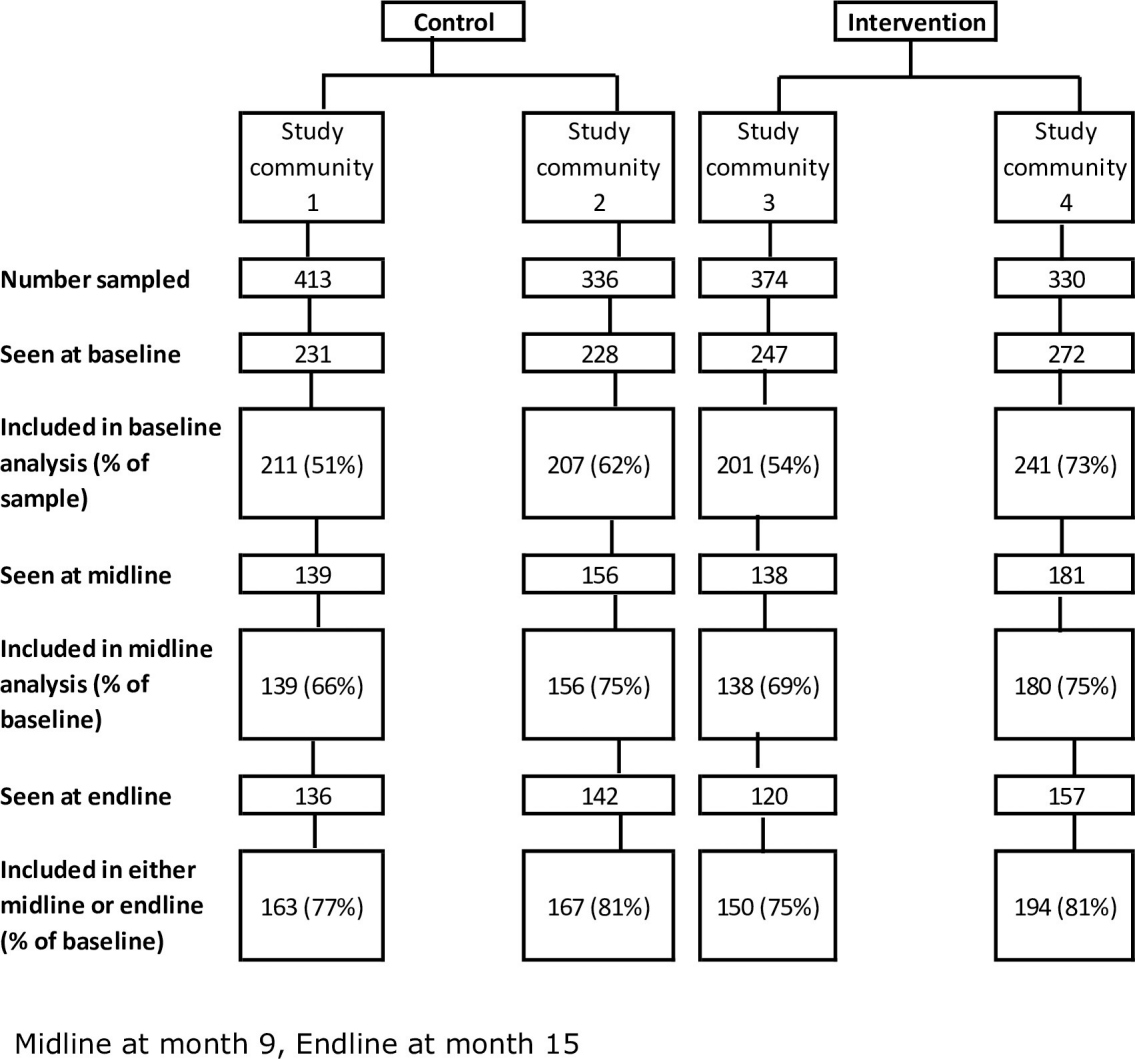
Consort flow.

The overall population size of the four fishing communities was 2989; of these, we enrolled 862 participants. We excluded 73 people from the baseline results as their age or sex was different to the census (>8-year age difference). The response rate at baseline, based on census data, was 62% in the control arm; after subsequent exclusions (see Fig 1), 56% of sampled participants are included in the baseline results. In the intervention arm 74% of sampled participants were included in the baseline survey; after exclusions, 63% were included in the baseline analysis. A total of 614 (63%) and 555 (57%) of enrolled participants were seen at midline and endline respectively (Fig 2). Contamination existed, 13 people were documented to have crossed from the control arm to the intervention arm. Three people crossed from the intervention arm to the control arm. The study was conducted at a time when displacements following land acquisition by private landowners, as well as migration associated with law enforcement to curb illegal fishing, were underway.

Table 1 shows key baseline characteristics of participants by study community and arm.

**Table 1:** Baseline characteristics of adults in a pilot HIV combination prevention community randomised control trial among fishing communities along the shores of Lake Victoria, Uganda (May 2014).

	Control villages		Intervention villages	
Outcome	Village 1	Village 2	Village 3	Village 4
**Included in baseline analysis**	211	207	201	241
**Setting**	Urban	Rural	Urban	Rural
**Sex**				
**Male**	108 / 211 (51.2%)	124 / 207 (59.9%)	81 / 201 (40.3%)	124 / 241 (51.5%)
**Female**	103 / 211 (48.8%)	83 / 207 (40.1%)	120 / 201 (59.7%)	117 / 241 (48.5%)
**Age group**				
**18–24**	66 / 211 (31.3%)	72 / 207 (34.8%)	88 / 201 (43.8%)	90 / 241 (37.3%)
**25–34**	80 / 211 (37.9%)	82 / 207 (39.6%)	63 / 201 (31.3%)	86 / 241 (35.7%)
**35+**	65 / 211 (30.8%)	53 / 207 (25.6%)	50 / 201 (24.9%)	65 / 241 (27.0%)
**Away for > one month in a year^‡^**				
**Yes**	52 / 208 (25.0%)	43 / 206 (20.9%)	48 / 200 (24.0%)	46 / 237 (19.4%)
**Education (183 missing values)**				
**Pre-primary school**	32 / 196 (16.3%)	17 / 132 (12.9%)	23 / 195 (11.8%)	15 / 155 (9.7%)
**Primary school**	99 / 196 (50.5%)	90 / 132 (68.2%)	85 / 195 (43.6%)	92 / 155 (59.4%)
**Secondary school**	55 / 196 (28.1%)	24 / 132 (18.2%)	62 / 195 (31.8%)	42 / 155 (27.1%)
**Higher or above**	10 / 196 (5.1%)	1 / 132 (0.8%)	25 / 195 (12.8%)	6 / 155 (3.9%)
**Currently married**				
**Yes**	175 / 211 (82.9%)	162 / 207 (78.3%)	157 / 201 (78.1%)	204 / 241 (84.6%)
HIV status*				
**Positive**	38 / 211 (18.0%)	38 / 207 (18.4%)	22 / 201 (10.9%)	40 / 241 (16.6%)
Sexual partners (last 3 months); Mean (SD)**				
**Mean (SD)**	1.21 (0.9)	1.14 (0.7)	1.19 (1.7)	1.19 (2.1)
**None; n/N (%)**	18 / 188 (9.6%)	16 / 189 (8.5%)	21 / 169 (12.4%)	22 / 218 (10.1%)
**Circumcised—men only^†^**				
**Yes**	37 / 102 (36.3%)	47 / 111 (42.3%)	50 / 78 (64.1%)	58 / 114 (50.9%)

‡ 9 missing values

‡‡ (183 missing values)

*4 missing values

**96 missing values

†32 missing values

### Process evaluation

We provided the study communities with the following services: 97,580 male condoms and 4280 female condoms; 3904 people received HCT, and 345 who tested positive were referred for ART. We provided VMMC to 650 men (83% of these were in the intervention arm), Table 2.

**Table 2:** Process evaluation for interventions.

Intervention delivered	Control	Intervention	Overall
**Condom promotion and distribution**	23,040 1070	74,540 3210	97,580 male condoms 4280 female condoms
**HCT visits**	1524	2380	3904
**Referral for ART**	135	210	345
**Number of male circumcisions performed**	109*	541	650

* Roll out of VMMC in the control sites coincided with changes in national policy and WHO VMMC and Tetanus vaccination guidelines. We provided 247 men in the control arm three doses of TT vaccination prior to VMMC, 54% were circumcised.

### Factors associated with loss to follow up (LTFU)

Overall LTFU was 21.6% and was similar by arm. In the control arm, LTFU was 22.8% in community 1 and 19.3% in community 2. In the intervention arm, it was 25.4% and 19.5% in the two communities respectively. Unadjusted odd ratios for LTFU are reported in Table 3. After adjustment, the strongest predictors of being lost to follow-up were having lived in the community for less than one year (aOR = 2.74; 95%CI:1.76–4.27), being HIV positive (aOR = 2.15; 95%CI:1.38–3.35), being young (18–24: aOR = 1.94; 95%CI:1.20–3.12, 25–34:aOR = 1.38; 95%CI:0.86–2.22, compared to being 35 or older), living in an urban area (aOR = 1.48; 95%CI: 1.05–2.09), being away from the area for more than one month per year (aOR = 1.63; 95%CI:1.10–2.42). Sex was not a strong predictor of being LTFU (aOR 1.08 for being female; 95% CI 0.76–1.55).

**Table 3:** Loss to follow-up (LTFU) of study participants from baseline to endline.

Characteristic	Total	Lost	Crude Odds Ratio of being LTFU (95% CI)
**Overall**	**860**	**186 (21.6%)**	
**Setting**			
**Urban**	412	99 (24.0%)	Baseline
**Rural**	448	87 (19.4%)	0.76 (0.55, 1.05)
**Sex**			
**Male**	437	83 (19.0%)	Baseline
**Female**	423	103 (24.3%)	1.37 (0.99, 1.90)
**Age group**			
**18–24**	316	88 (27.8%)	Baseline
**25–34**	311	64 (20.6%)	0.67 (0.46, 0.97)
**35+**	233	34 (14.6%)	0.44 (0.29, 0.69)
**Away for > one month in a year**			
**Yes**	189	52 (27.5%)	Baseline
**No**	662	132 (19.9%)	0.66 (0.45, 0.95)
**Time lived in community**			
**Less than a year**	117	47 (40.2%)	Baseline
**More than a year**	743	139 (18.7%)	0.34 (0.23, 0.52)
**Education**			
**Pre-primary school**	87	17 (19.5%)	0.84 (0.47, 1.51)
**Primary school**	366	82 (22.4%)	Baseline
**Secondary school**	183	49 (26.8%)	1.27 (0.84, 1.91)
**Higher or above**	42	9 (21.4%)	0.94 (0.43, 2.05)
**Fishing industry**			
**No**	551	130 (23.6%)	Baseline
**Yes**	309	56 (18.1%)	0.72 (0.51, 1.02)
**Farmer**			
**No**	639	149 (23.3%)	Baseline
**Yes**	221	37 (16.7%)	0.66 (0.44, 0.98)
**Hotel /restaurant/bar**			
**No**	782	159 (20.3%)	Baseline
**Yes**	78	27 (34.6%)	2.07 (1.26, 3.41)
**Currently married**			
**No**	162	41 (25.3%)	Baseline
**Yes**	698	145 (20.8%)	0.77 (0.52, 1.15)
**HIV status**			
**Negative**	722	143 (19.8%)	Baseline
**Positive**	138	43 (31.2%)	1.83 (1.22, 2.75)
**No partners in last 3 months**			
**At least one**	687	144 (21.0%)	Baseline
**None**	77	16 (20.8%)	0.99 (0.55, 1.77)
**Male circumcised**			
**No**	214	45 (21.0%)	Baseline
**Yes**	191	38 (19.9%)	0.93 (0.57, 1.51)
**Study arm**			
**Control**	418	88 (21.1%)	Baseline
**Intervention**	442	98 (22.2%)	1.07 (0.77, 1.48)

### Uptake of single and combined methods

In Table 4, self-reported abstinence/faithfulness (i.e. had no sex or had sex with one regular partner in the last three months) increased between baseline and endline in both arms, from 53% to 73% in the control arm, and 55% to 67% in the intervention arm. Reported condom use throughout the study period was 36% in the intervention arm compared to 28% in the control arm. There was a 20% increase in the number of male participants in the intervention arm reporting VMMC compared to a 7% increase in the control arm.

**Table 4:** Uptake of HIV prevention methods.

Outcome	Control villages		Intervention villages	
	Village 1	Village 2	Village 3	Village 4
**Condom use**				
Baseline	92 (43.6%)	89 (43.0%)	87 (43.3%)	88 (36.5%)
Round 2	36 (25.9%)	48 (30.8%)	58 (42.0%)	54 (30.0%)
Round 3	30 (22.1%)	49 (34.5%)	44 (36.7%)	54 (34.8%)
**Abstain or faithful**				
Baseline	118 (55.9%)	103 (49.8%)	105 (52.2%)	140 (58.1%)
Round 2	99 (71.2%)	95 (60.9%)	80 (58.0%)	115 (63.9%)
Round 3	104 (76.5%)	98 (69.0%)	82 (68.3%)	101 (65.2%)
**Male circumcised**				
Baseline	37 (36.3%)	47 (42.3%)	50 (64.1%)	58 (50.9%)
Round 2	27 (40.3%)	47 (51.1%)	46 (79.3%)	60 (66.7%)
Round 3	21 (34.4%)	43 (56.6%)	45 (86.5%)	50 (70.4%)
**HIV test ever**				
Baseline	175 (83.7%)	180 (87.8%)	172 (86.4%)	207 (86.3%)
Round 2	137 (100.0%)	155 (100.0%)	137 (100.0%)	175 (100.0%)
Round 3	131 (99.2%)	138 (100.0%)	118 (100.0%)	152 (100.0%)

## Discussion

Fishing communities are an important population for HIV transmission in Uganda. We demonstrate that implementation of HIV combination prevention in this highly mobile hyper-endemic community is practically feasible, although not without challenges. High rates of non-participation and poor retention may hinder evaluation of population level impact of combination prevention on HIV incidence in high prevalence settings.

Compared to the general population, fishing communities are at high-risk of HIV, and are a bridge population for HIV transmission. As such, they are a priority population for combination prevention. It is reassuring that, in this small sample, the observed change was in the direction we had hoped for. However, our findings suggest that delivery of combination prevention interventions remains challenging, and achieving high levels of adherence could be even more challenging. Effective HIV prevention needs to take into account underlying socio-cultural, economic, political, legal and other contextual factors.

Recent findings from a large open cohort study in Rakai, South western Uganda (n = 1823 HIV-negative people, contributing 5188 person years) in one fishing community on Lake Victoria reported moderate LTFU (36%), over a 4 year period, from November 2011 to February 2016, compared to LTFU of 21.6% in our cohort, over a 2 year period, from July 2014 to February 2016. In addition, between 2011 and 2017, ART coverage in the Rakai cohort increased from 19% (95%CI: 16–22%) to 81% (95%CI: 75–87%), and male circumcision coverage increased among all men from 39% (95%CI: 35–42%) to 63% (95%CI: 59–67%), achieving 54% population-level HIV viral suppression and 58% reduction in HIV incidence [6]. It might be the case that the Rakai cohort was part of is an established long-term longitudinal cohort-the open population based Rakai community cohort, offering free healthcare services, and with better capacity to monitor and address retention problems, over time.

In our study, the refusal rate and loss to follow-up (LTFU) were high. Some have suggested that > 20% LTFU may pose serious threats to validity [32]. LTFU could have been facilitated by contextual factors including rampant displacements following land acquisitions by private landowners, as well as migration as noted above. Loss to follow up in our study was independently associated with being female, living with HIV and working in the hospitality industry. This highlights the additional need for female-initiated HIV prevention products, and possibly, for interventions aimed at improving retention within the hospitality industry. However, it is possible that people living with HIV might have enrolled into HIV care prior to commencement of the trial reducing their motivation to continue participating in the HIV prevention trial.

Overall, local beliefs such as condomless sex with a casual partner to cast away spells and the ‘wound healing power’ of vaginal fluids and practices may have influenced uptake and potentially, the effectiveness of VMMC [28]. In addition, owing to changes in WHO guidelines during the course of the study, the introduction of TT vaccination prior to VMMC, to mitigate the risk of tetanus infection, could have further reduced VMMC uptake.

Our study is one of few studies investigating feasibility of HIV combination prevention in fishing communities in the East African great lakes region. However, a limitation of the study is that as it was a pilot study with only four communities, we were unable to take into account the effect of clustering of households within communities in the analyses. For this reason, it is not possible to carry out formal hypothesis tests for any difference between intervention and control communities, nor for a difference between rural and urban communities. Further, the study was not powered for formal hypothesis testing for effectiveness or efficacy of the intervention. Therefore, estimates of uptake may be imprecise and must be interpreted with caution. We actively referred HIV positive participants for ART but did not monitor uptake of referral and ART initiation through the ART providers. We did not objectively assess acceptability of specific interventions but participants found VMMC interesting and acceptable, as we have found elsewhere [28]. Finally, the results may lack representativeness of the estimated 72 fishing communities in the three study districts, and because of the small sample size and number of clusters, and the fact that we worked on landing sites alone, excluding islands.

Data on sexual behavior and uptake of HIV prevention methods was by self-report. This might have resulted in error in self-report measures, due to social desirability [33]. It is possible that participants in the intervention arm over-reporting uptake of HIV prevention methods, which may have over-estimated the difference in uptake between arms.

We did not document process evaluation data regarding number of prevention products or services delivered at community level by either public or private providers, in the control arm.

Follow-up time was relatively long for a highly mobile population. This might have increased the possibility of overlapping of cluster membership. In our study, we did not objectively monitor change of cluster membership. These discrepancies might have occurred due to errors in data collection, as we did not use electronic capture for both the census and baseline data collection, or impersonation, or the same person giving different answers.

The high rates of LTFU and low participation in this mobile population confirms the need to examine approaches to community engagement and to trial modes of delivery of HIV prevention before implementing HIV combination prevention packages this could improve uptake of methods and potentially study retention. Monitoring and documentation of co-enrolment to minimise recruitment bias, and contamination across study communities using biometric scanning technology would be of benefit. Previous work has highlighted the role of fingerprint-based biometric technology, to (i) identify potential trial participants in Vietnam [34], tracking mobility of pastoralists in Chad [35], and to (iii) link health facility data with demographic surveillance data in South Africa [36].

We provided tetanus vaccination to men prior to VMMC, as per the recent WHO guidelines [30]. Providing tetanus vaccination alongside VMMC was acceptable and feasible but quite challenging. This is mainly because in a highly mobile setting, it is difficult to maintain high repeat visits for second and thirds TTC vaccinations. This has implications for uptake of VMMC.

Finally, an important source of heterogeneity in uptake of interventions could have arisen from the size and nature i.e. age, gender and baseline HIV prevalence of the study communities. However, we did not assess for this in the pilot because the number of clusters was too small. The design of a future effectiveness trial would need to allow for between-cluster heterogeneity.

## Conclusions

Recruitment and retention of study participants in longitudinal trials in highly mobile fishing communities remains challenging. Improving recruitment and retention and investigating modes for delivering and supporting delivery of HIV combination prevention should be a primary focus of future trials. Our findings have implications for HIV prevention, treatment and care programmatic planning for similar communities at risk of HIV infection.
